# The State of Atrial Fibrillation in 2020

**DOI:** 10.19102/icrm.2021.120105

**Published:** 2021-01-15

**Authors:** Rahul N Doshi

**Affiliations:** ^1^Cardiovascular Center of Excellence, HonorHealth, Cardiac Arrhythmia Group, HonorHealth Medical Group, Scottsdale, AZ 85251, USA

**Keywords:** Ablation, atrial fibrillation, COVID-19, electroporation

“*Human sacrifice, dogs and cats living together, mass hysteria!”—Peter Venkman, Ghostbusters.*

This past year seems unlike any we have experienced in recent history and, like many of you, I would like a do-over. Despite the global pandemic, political unrest, a country divided, and racial inequalities (to name a few), I would remind everyone that the medical community has continued to treat, educate, and unify. The editors of *The New England Journal of Medicine* made history by taking a political stand for the first time in 208 years!^1^ In these unprecedented times, continuing to focus on the science and innovation may be even more important in both guiding and unifying people. Despite the challenges, including virtual meetings replacing onsite scientific sessions, this past academic year was rich with significant clinical advancements regarding the cornerstone of every electrophysiologist’s clinical practice: the management of patients with atrial fibrillation (AF).

## COVID-19 and atrial fibrillation

It seems only fitting that we begin with severe acute respiratory syndrome coronavirus 2 infection and its association with AF. Certainly, multiple reports regarding myocardial injury^2^ and the high incidence of myopericarditis even in patients convalescing from coronavirus disease 2019 (COVID-19) have been published.^3^ Given the established association of pericarditis and AF, it seems only logical that there would be an increased incidence of atrial arrhythmias associated with COVID-19. Bhatla et al. at the University of Pennsylvania reviewed their single-center incidence of dysrhythmias in patients hospitalized for COVID-19^4^ and identified 25 patients with new-onset AF amongst their 700-patient cohort. However, the authors concluded that the new arrhythmias were associated with the severity of systemic illness as stratified by intensive care unit admission rather than necessarily related to the occurrence of viral infection itself.

Recently, Abrams et al. reported characteristics associated with mortalities during the initial phase of the pandemic in New York City.^5^ Of the 133 patients among 1,258 total study participants who died, 14.3% presented with AF and an additional 10.1% subsequently developed AF during their hospital stay. This high incidence of AF in patients who ultimately experienced poor outcomes is consistent with reports suggesting that cardiac involvement in disease in general is associated with a poor prognosis.^2^ In a separate publication, Peltzer et al. examined the incidence of AF and the correlation with outcomes amongst patients with severe respiratory distress syndrome, also in New York City.^6^ In a cohort of 1,053 patients, 18.4% had AF or atrial flutter. Of these, 9.6% of cases involved new-onset disease. Patients with AF had increased levels of biomarkers of heart failure and inflammation as compared with those without and a much higher mortality rate (39.2% with AF vs. 13.4% without; p < 0.001). New AF/atrial flutter was associated with an odds ratio of 2.87 (p < 0.001) for mortality in this patient group. These results demonstrate that AF is independently associated with an increased risk of death in patients ill with COVID-19 and is associated with increased markers of heart failure and inflammation. Of course, what is not known is whether or not aggressive treatment of AF in these patients may significantly affect or improve their overall prognosis.

Another facet of the COVID-19 pandemic and the treatment of AF regards performing procedures that are generally considered elective. Early on, the Heart Rhythm Society Task Force provided some guidance as to what type of procedures should be considered urgent rather than elective, with the latter suggested to be delayed—at least in the initial stages of the pandemic.^7^ Urgent procedures, according to the published guidelines, included ablation for incessant hemodynamically unstable or poorly tolerated AF or atrial flutter and cardioversion for these rhythms. AF ablation in stable patients without heart failure was considered elective or nonurgent and the decision to proceed with this procedure was rendered dependent upon the local state of the pandemic, as we all experienced directly. It is expected that this stratification of procedural necessity will continue through subsequent waves and possibly during future pandemics. Further, increased utilization of ambulatory diagnostics and virtual telehealth visits may be increasingly adopted in similar forthcoming scenarios in centers specializing in the treatment of AF.

## EAST-AFNET 4 trial

Move over, AF Follow-up Investigation of Rhythm Management (AFFIRM) trial—the Early Treatment of Atrial Fibrillation for Stroke Prevention Trial (EAST-AFNET 4) trial is, by many estimates, potentially the most important landmark clinical trial in the field of cardiac electrophysiology to have emerged this year. The trial, presented during the virtual European Society Congress and published recently, was an international multicenter prospective randomized open-label trial in patients with a recent (< 1 year) diagnosis of AF and certain cardiovascular conditions (including older age) who were randomized to symptom-only control or a rhythm-control strategy (ie, antiarrhythmics or ablation).^8^ The primary composite endpoint was cardiovascular death or hospitalization for stroke, heart failure, or acute coronary syndrome. Two thousand seven hundred eighty-nine patients were randomized and followed for a mean of 5.1 years. The trial was stopped after reaching its efficacy endpoint. Patients treated with rhythm control reached the primary endpoint at 3.9/100 patient-years versus 5.0/100 patient-years in the usual treatment arm (hazard ratio: 0.79; p = 0.005). The rhythm-control strategy was most commonly antiarrhythmic drug therapy in 86.8% initially (with 50% treated with propafenone) but, by two years, almost 40% had undergone catheter ablation.

The primary endpoint was, most commonly, heart failure hospitalization, although this scenario still accounted for less than half of the total events. Patients with rhythm control were more likely to be in sinus rhythm (82.1% vs. 60.5%), suggestive of constituting a “less sick” population. Moreover, roughly three-quarters of patients in the trial were asymptomatic.

This is the first trial to demonstrate that introducing a rhythm-control strategy early on in the diagnosis of AF is associated with improved outcomes measured as hard clinical endpoints in a largely asymptomatic population. The EAST-AFNET 4 trial enrolled a very similar demographic to that of the AFFIRM trial, with participants having almost identical ages and comorbidities as well as timing of the first episode of AF.^9^ However, this trial differs from the AFFIRM trial and other such investigations in that it included catheter ablation as a rhythm-control treatment strategy. In addition, the most common antiarrhythmic drug used in the AFFIRM trial was amiodarone.

The EAST-AFNET 4 trial clearly supports the on-treatment analysis for the Catheter Ablation Versus Antiarrhythmic Drug Therapy for AF (CABANA) trial,^10^ as we continue to move toward pursuing earlier treatment for AF, including by employing an ablation strategy for rhythm control.

## Electroporation

Certainly, pulsed-field ablation or electroporation has recently generated the most buzz in the area of AF ablation—with good reason. Multiple feasibility studies using both novel and current technologies to develop monophasic or biphasic waveforms have been presented, creating excitement surrounding this energy source. With this ablation protocol, energy is rapidly delivered with good tissue specificity, thus potentially eliminating the risk of collateral damage that remains an inherent risk with the use of traditional energy sources such as radiofrequency energy. Two featured presentations during the virtual Heart Rhythm Society 2020 Science late-breaking clinical trial sessions introduced two different platforms for ablation with electroporation in AF. Reddy et al. previously presented first-in-man data concerning the treatment of paroxysmal AF with pulmonary vein isolation (PVI).^11,12^

More recently, results from the Feasibility Study of the FARAPULSE Endocardial Multiablation System in the Treatment of Persistent AF (PersAFOne) demonstrating the benefits of pulsed-field ablation employing both a pulmonary vein strategy and a posterior wall ablation strategy for the treatment of persistent AF were published.^13^ Posterior wall ablation/isolation increases the success rates for ablation for persistent AF^14^ but, certainly, a limitation to the widespread adoption of this approach remains the potential for esophageal injury with traditional energy sources. The study was a single-arm study using a multispline catheter and biphasic bipolar pulsed-field ablation (Farapulse Inc., Menlo Park, CA, USA). In 25 patients, acute PVI and posterior wall isolation were achieved in all cases, together with success for all 13 patients who underwent cavotricuspid isthmus ablation. The procedure was very rapid, with a mean time for PVI of 22 minutes and that for posterior wall ablation of 10 minutes. In addition, 21 patients underwent esophagogastroduodenoscopy and no mucosal lesions were identified.

From these results, it is clear technologies such as electroporation demonstrate both improved efficiency and safety, while investigations such as EAST-AF support that an earlier rhythm-control strategy results in improved outcomes. Taken together, the presented findings could potentially drive increased use of catheter ablation for AF.

## Non–pulmonary vein ablation and mechanisms for persistent atrial fibrillation

There has long been an interest in “uncovering” mechanisms for the maintenance of persistent AF. Historically, complex fractionated atrial electrogram mapping used the appearance and estimated frequency of local electrograms as a surrogate for either focal activity, rotational activity, or slow conduction (without really elucidating a mechanism).^15^ Focal impulse and rotor modulation or rotor mapping and other technologies sought to elucidate an ideal mechanistic approach for an individual patient with AF and were intellectually attractive, with initial data suggesting success with freedom from AF,^16^ although these results have not been widely reproduced.

Recently, the Utilizing Novel Dipole Density Capabilities to Objectively Visualize the Etiology of Rhythms in AF (UNCOVER-AF) trial was published,^17^ which assessed the use of a novel noncontact mapping system that employs ultrasound and diode ionic activation/charge density to create whole-chamber activation maps (Acutus Medical, Carlsbad, CA, USA). The trial was a multicenter, nonrandomized study examining the 12-month efficacy of catheter ablation for AF in 129 patients. The treatment plan was to use patterns of activation incorporated into PVI and then to approach the remaining areas of abnormal activation (rotational activity or slow conduction) with ablation connected to an anatomic obstacle. Willems et al. reported very high first (72.5%) and second (93.2%) one-year efficacy rates with catheter ablation.^17^ These authors also localized areas of abnormal activation and reported some consistent patterns appeared to be occurring more frequently in the study population, although they were not necessarily present in each individual patient. Targeting at least two of three activation patterns did correlate with a higher likelihood of AF-free survival (odds ratio: 2.84; p = 0.02).

While these data need to be interpreted with caution given the lack of a control arm, this technology, if nothing else, constitutes a novel approach by which to apply a mechanistic guide to catheter ablation for persistent AF beyond pulmonary venous triggers.

## Conclusions

It is clear from the study results announced to date that we as the AF-treating community may be faced with an interesting dilemma when employing treatment strategies. We may, someday in the future, be able to characterize patient-specific mechanisms for AF in a manner enabling us to perform less empiric ablation. However, this may be practically challenged as technologies such as pulsed-field ablation may make it easier to employ anatomic-based strategies that might be considered empiric but more efficient and without additional safety concerns. This dilemma might be very relevant as we start treating patients even earlier in their disease course, as the EAST-AF trial would suggest. Finally, the long-term sequelae of COVID-19 remain to be elucidated, with additional data needed; depending on the likelihood of developing AF after recovery, the prevalence of AF may drastically increase, placing a greater burden on the cardiac electrophysiology community.
